# Prevalence and Patterns of Cognitive Impairment in a Sample of Community Dwelling Older People in Nigeria

**DOI:** 10.14283/jarlife.2023.15

**Published:** 2023-11-16

**Authors:** V. Ucheagwu, B. Giordani

**Affiliations:** 1 Nnamdi Azikiwe University Nigeria Nigeria; 2 University of Michigan USA Michigan, USA

**Keywords:** Dementia, prevalence, Nigeria, older adults, neuropsychological assessment

## Abstract

**Objective:**

Prevalence and patterns of cognitive impairment were studied in older people from Nigeria.

**Method:**

Four hundred and forty one participants (263 females; age: 60-87) were recruited from community dwelling adults in Anambra state Nigeria. Five domains of cognition were tested using the Uniform Data Set Version 3 (UDS-3).

**Result:**

Prevalence: 49.7% were classified as normal cognition, 34% as borderline, 12.9% as MCI (2.72% with amnesic MCI) and 3.4% as dementia. We showed in descending order in that 13% of the participants were impaired on visual-spatial index; 6.8% on memory index; 5.2% on attention/concentration index; 2.7% were impaired on executive function index and 34.80% (based on mean) of the participants were impaired on processing speed index. There were significant interaction effects for gender and education on visual spatial and attention domains respectively. Significant effects of education were seen on executive function and processing speed while interaction effect was found on executive function alone. 8% scored 1.5 SD below the mean on MoCA. There was a significant effect of education on MoCA with the pairwise comparison showing a significant difference between tertiary education and other two levels of education. The groups did differ significantly for hypertension on MoCA.

**Conclusion:**

This study showed a high prevalence of cognitive impairment among older adult population from Nigeria. A significant proportion of the sample were impaired on the visual spatial domain and at least half of the participants were impaired on one cognitive domain. Hypertensive participants performed significantly poor on MoCA compared to non-hypertensive group.

## Introduction

**I**n a classic article by Folstein and colleagues (1), cognitive impairment (CI) was defined as a diminished capacity to know the world which could result from many clinical disorders like dementia, mental retardation, aphasia and traumatic brain injury. CI cuts across age spectrums but is most likely seen in older adults as its prevalence increases with aging. Changes in cognition occur as part of the aging process but could form a clinical disorder to the extent that it becomes debilitating and affecting individuals’ functioning. Central to this diagnostic scheme is the clinical construct of mild cognitive impairment (MCI) (2-3) that is generally regarded as the borderland between the cognitive changes of aging and very early dementia (4). In other words, older adults could either show cognitive changes related to aging or graded abnormal cognitive related changes from mild to severe forms of cognitive impairment. CI in older people could progress to dementia particularly dementia of Alzheimer’s type (DAT) depending on the severity. The major risk factor for dementia is age, with the prevalence doubling every 5 years after the age of 65 and stabilizing to around 50% in the 8th decade (5-7). The annual rate in which MCI progresses to dementia varies between 8% and 15% per year, indicating that it is an important condition to identify and treat (4). However, it must be noted that not all CI represent incipient Alzheimer’s disease nor did all patients with CI have just a memory impairment. Cognitive impairment could occur in either domains of cognitive functions like memory, attention/concentration, language, executive and visuospatial functions.

The prevalence of CI in Nigeria is less studied than in high income countries. In a survey of cognitive impairment among Yoruba speaking sample from Ibadan Nigeria, 152 (62%) out of 423 individuals studied were diagnosed with cognitive impairment no dementia (CIND) while 28 (6.61%) were diagnosed with dementia (7). In northern Nigeria (8), survey of 323 older adults showed dementia prevalence at 2.79% (CI 1– 4.58%) representing 66.67% of all the cases of dementia in the sample. In south-west Nigeria, 10.1% prevalence of probable dementia were found (9) using the 10 Word Delay Recall test adapted from Consortium to Establish a Registry for Alzheimer’s Disease CERAD (10) . In the North Central Nigeria, Ochayi and Thatcher (11) using the Community Screening Instrument for Dementia (CSID), showed a 6.4% overall prevalence of dementia and in south east Nigeria, 23.1% depression prevalence was shown in older adult sample with 20.7% complaining of forgetfulness (12).

Although there are few studies that have examined cognitive impairment in Nigeria, these studies have two major limitations. First, none of the studies used a comprehensive neuropsychological battery to characterize the domains of CI. The majority of the studies utilized short mental status tests that represented measures of global cognition while others used a single test to determine dementia. This methodology could lead to several problems. For instance, shortening a test and detaching it from its standardized administration, scoring, and normative referencing may make it less sensitive or reliable violating the statistical maxim that multiple measures provide a more reliable estimate of a cognitive construct than any single measure (13). Also, the lack of objective tests from other cognitive domains reduces the ability to detect cognitive impairment profiles that might identify distinct subtypes of CI that vary in clinical and biological characteristics (14-15). Second, the studies in Nigeria did not show the domains of cognitive impairment that were most affected in the ageing population. Similarly, they were not able to clearly characterize graded levels of CI (from normal cognitive ageing to severe CI) in the participants based on the number of cognitive domains and or patterns of cognitive impairment (amnestic and non-amnestic) observed.

The present study aimed to examine the prevalence and patterns of cognitive impairments using actuarial neuropsychological test batteries that tap 5 major domains of cognition. These could be the best way to start estimating the prevalence of CI in the Nigerian ageing population and provide a clear clinical picture of the patterns of cognitive decline and a more comprehensive understanding of people at risk for AD and dementia. Also, we examined the association of some modifiable risk factors and demographics on cognitive decline. We hypothesized that a greater percentage of older adults would perform poorly on memory and attention domains relative to other domains. We further hypothesized significant differences respectively in education, gender, diabetes and hypertension on cognitive performance.

## Method

### Participants

Four hundred and forty one older adults (263 females; age: 65-87; mean: 67.99; SD : 3.67) participated in the study. Participants were recruited from the adult population in Ukpo, Dunukofia local government area of Anambra state. Participants were participating in longitudinal cohort study for blood based biomarkers and dementia in Nigeria (See procedure section for details). One hundred and twelve had tertiary education, 104 had secondary education, while 165 had primary and 109 had no formal education. Our university clinic in the area is running a cognitive screen for every healthy adult over age 65 using the door to door knocking approach and clinical visits. The cognitive screening further involves measures of metabolic syndrome including lipids, diabetes and hypertension.

### Instrument

#### Neuropsychological test battery

The Uniform Data Set-Version 3 (UDS-3) (2015) created and published by the Alzheimer’s Disease Center Clinical Task Force of the National Alzheimer’s Co-ordinating Center United States was used for cognitive testing. UDS-3 measures domains of cognition using selected cognitive tests. For the present study, 5 cognitive domains were assessed using the following tests : attention/ concentration (number span test: forward and backward), memory (craft story immediate and delayed (16), visuo-spatial (Benson design: immediate and delayed), processing speed (TMT: A & B) and executive function (category fluency: animal and vegetable; control oral word: FAS). Among the UDS tests used, all were non-verbal except the craft story test (CST). CST was a story test written in English language describing a story of young boy Ricky playing football. CST was translated to the participants’ language (Igbo language) and back translated to English by 2 experts in Igbo and English languages respectively. The two forms were administered to 150 older adults to determine the level of agreement between the forms. The correlation of 0.89 was obtained between the two forms. For full details of the UDS -3 battery see National Alzheimer’s Co-ordinating Center University of Washington. Montreal Cognitive Assessment (MoCA) (17) was used to measure global cognition in the participants. MoCA is a widely used screening assessment for detecting cognitive impairment and has been used in a similar cohort in Nigeria (18). In addition to the cognitive measures, depression status was assessed using the Geriatric Depression Scale (GDS) (19).

### Procedure

Participants were recruited through local community advertisement at faith-based organisations, elderly retirement groups and town criers within Ukpo town in the Dunukofia local government area. Interested participants were invited to the community health care center for initial history taking and informed consent. The neuropsychological test battery was administered to participants on second visit. The study was carried out in accordance with the Helsinki Declaration on human participants’ involvement in research studies and ethical approval (IRB) granted by Nnamdi Azikiwe University Ethical Review Board.

### Design and Statistics

The study was a longitudinal study to evaluate AD blood based-biomarkers and cognition in older adults (Alzheimer’s Disease Biomarker-Nigeria). The present report was the baseline data on cognition collected at baseline (year 1). Descriptive statistics and multiple analysis of variance were used for data analysis. We developed a measure of impairment called: Cognitive impairment severity index (CISI) by classifying participants’ performance into normal cognition (NC), borderline cognition (BC), mild cognitive impairment (MCI), and severe cognitive impairment (SCI). This was developed by summing up the performance of participants on the 5 cognitive domains. Cut off score of 1.5 SD below the mean (using sample norms) was used as a measure of impairment on that domain. Hence, participants were classified in normal cognition (NC) category if they score did not score below the cut-off on any of the domains. Borderline cognition (BC) were participants that scored below the cut-off on only 1 domain. MCI group was classified as participants that scored below the cut-off on 2 domains while dementia group was classified as participants that scored below the cut-off on 3 or more cognitive domains. This model represents actuarial neuropsychological criteria for MCI and dementia diagnoses (20-21). We calculated percentages for the means and SDs of the 5 cognitive domains, CISI and UDS subscales to demonstrate the prevalence of cognitive impairments in our sample. Multivariate analysis of variance was used to determine the roles of independent variables: education, gender, depression and metabolic syndrome (hypertension and diabetes) on 5 cognitive domains. The independent variables were nominally scaled (yes/no) based on self-report. The norms for the study were derived from the sample data.

## Results

The results of participants’ neuropsychological assessment are presented below. First we presented their performances based on 5 cognitive domains. Then we presented results showing participants’ cognitive impairment severity index (CISI) using Jack and Bondi method (20-21). CISI was classified as follows: normal cognition (participants that performed above 1.5 SD on all 5 cognitive domains), borderline impairment (participants that performed below 1.5 SD on one cognitive domain), mild impairment (participants with less than 1.5 SD on two domains) and severe cognitive impairment (those with less than 1.5 SD on more than 2 cognitive domains).

**Figure 1.**
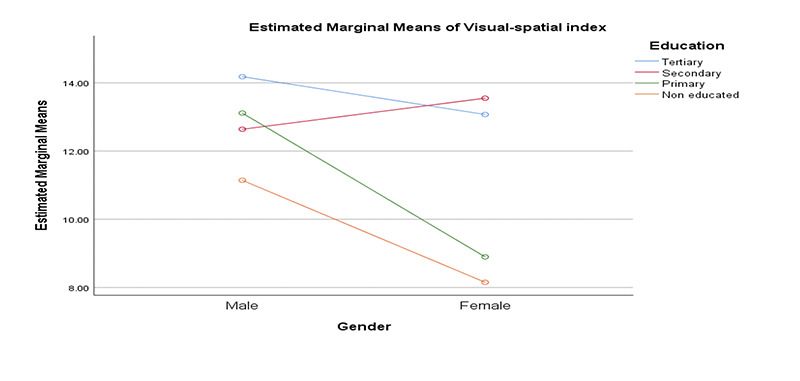
Interaction of gender and education on visual-spatial index

Table 1 shows the mean and standard deviation score of the participants on five cognitive domains and percentages of participants that performed below mean and SD scores respectively. At 1.5 SD level, participants performed worst on the visuo-spatial domain followed by the memory domain. While using a mean cut point, they performed more poorly on the processing speed followed by executive function domain.

**Table 1.** Mean, SD and Percentage of Participants that Scored below the Mean and SD on Cognitive Domains

Cognitive Domains	Mean	% Mean^a^ (N)	SD	%1 SD^b^	%1.5 SD^c^ (N)
Attention/Concentration	19.67	49.20 (217)	7.33	14.70	5.20 (52)
Memory	39.37	48.70 (215)	20.97	17.50	6.80 (68)
Visuo-spatial	11.72	41.40 (183)	6.08	17.60	6.80 (68)
Processing Speed	4.61	64.40 (284)	6.15		
Executive Function	31.07	56.50 (249)	13.74	18.00	27.0 (27)
MoCA	20.51	37.00 (163)	4.33	16.50	8.0 (79)

Note: a--- Percentage of participants that fell below the mean score on each cognitive domain; b--- Percentage of participants that fell below 1 SD from the mean on each cognitive domain; c--- Percentage of participants that fell below 1.5 SD from the mean on each cognitive domain.

Table 2 shows the number of participants that were characterized on the cognition impairment severity index.

**Table 2.** Frequency and Percentage of Participants on Cognitive Impairment Severity Index

Cognitive Impairments	N	Percentage (%)
Normal Cognition	219	49.70
Borderline	150	34.00
MCI	57	12.90
aMCI	12	21.05
Non aMCI	45	78.95
Severe Impairment	15	3.40

Note: MCI--- Mild cognitive impairment; aMCI--- Amnestic mild cognitive impairment.

We performed series of multivariate analysis to determine the roles of gender, education and depression on five cognitive domains. Our findings showed significant differences between gender and education on visual spatial and attention domains: Visual spatial; Gender: F (1,398) = 6.37 (ES: 0.02; Mean: Male = 13.20, Female = 8.15); Education: F (3,398) = 6.70 (ES: 0.05; Mean: Tertiary = 13.60, Secondary = 13.04, Primary = 10.89, Non-educated = 8.44); attention; Gender: Gender: F (1,398) = 5.52 (ES: 0.01; Mean: Male = 21.61, Female = 18.81); Education: F (3,398) = 23.57 (ES: 0.15; Mean: Tertiary = 24.47, Secondary = 20.58, Primary = 18.82, Non-educated = 14.93). There were significant interaction effects of gender and education on visual spatial: F (3,398) = 3.95 (ES: 0.03) and attention: F (3,398) = 3.52 (ES: 0.03).

**Table 3.** Mean, SD and Percentage of Participants that Scored below the Mean on UDS Measures

Cognitive Domains	Mean	% Mean^a^	SD	%1SD^b^	%1.5SD^c^
Craft Story Immediate	21.11	49.10	10.61	20.70	7.70
Craft Story Delayed	18.19	45.60	11.32	19.30	9.30
Benson Design Immediate	9.55	43.80	4.97	18.60	13.90
Benson Design Delayed	2.01	76.70	3.14		
Number Span Forward TL	6.69	51.70	2.43	2.43	7.99
Number Span Forward LS	5.73	47.80	3.09	13.40	9.13
Total Number Span Forward	12.43	51.70	4.66	7.77	4.50
Number Span Backward TL	4.06	59.90	2.57	13.80	10.50
Number Span Backward LS	3.17	58.30	1.61	9.80	9.80
Total Number Span Backward	7.20	58.00	4.08	13.60	9.80
TMT A (Error)	1.42	77.70	3.10		
TMT B (Error)	3.18	68.30	4.60		
Category Fluency (Animal)	8.91	50.70	3.51	13.80	3.60
Category Fluency (Vegetable)	6.43	55.30	2.65	9.80	3.20
Total Category Fluency	15.36	55.30	5.20	16.10	4.80
Control Oral Word	15.65	55.55	11.06	15.20	

Note: a--- Percentage of participants that fell below the mean score on each UDS measure; b--- Percentage of participants that fell below 1 SD from the mean on each UDS measure; c--- Percentage of participants that fell below 1.5 SD from the mean on each UDS measure.

**Figure 2.**
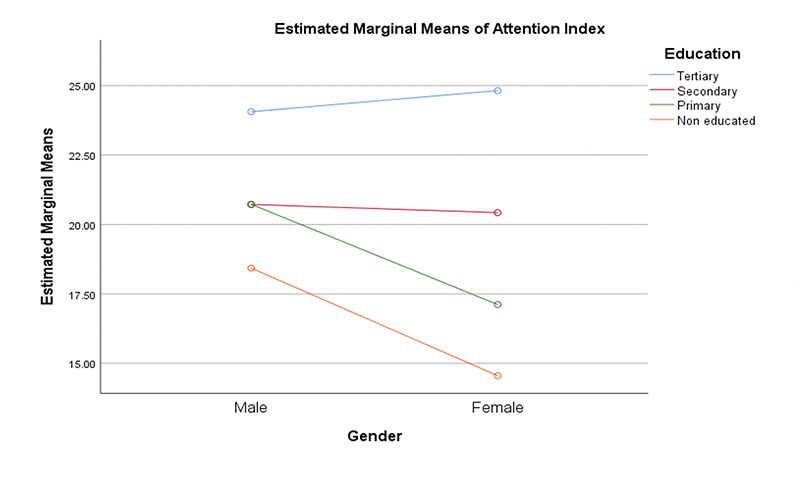
Interaction of gender and education on attention index

We found no significant effect of gender on executive function: F (1,398) = 1.90 (ES: 0.01) and processing speed: F (1,398) = 0.001 (ES: 0.001). Significant effects of education were seen on executive function: F (3,398) = 39.87 (ES: 0.23; Mean: Tertiary = 42.08, Secondary = 33.51, Primary = 29.36, Non-educated = 19.88) and processing speed: F (3,398) = 6.89 (ES: 0.05; Mean: Tertiary = 4.13, Secondary = 2.36, Primary = 5.58, Non-educated = 6.16). Significant interaction effect of gender and education was found on executive function index: F (3, 399) = 6.25 (ES: 0.05). Our result showed no significant difference of gender and education on memory index; Gender: F (1,398) = 0.43 (ES: 0.01); Education: F (3,398) = 2.17 (ES: 0.01; Mean: Tertiary = 43.54, Secondary = 40.90, Primary = 39.12, Non-educated = 33.22). Although no significant difference was found among levels of education, multiple comparisons using the scheffe method showed significant difference for tertiary education as compared with non-educated level. We found no significant difference of histories of hypertension and diabetes on processing speed and visual-spatial domains; Hypertension: Processing speed: F (1,398) = 0.11 (ES:0.001); visual-spatial index: F (1,398) = 2.50 (ES: 0.006)); diabetes: Processing speed: F (1,398) = 0.61 (ES:0.002); visual-spatial index: F (1,398) = 0.55 (ES: 0.001)). No interaction effects were found.

Equally no significant differences were seen on hypertension and diabetes on the attention and executive function domains; hypertension: attention: F (1,398) = 0.03 (ES:0.001); executive function: F (1,398) = 0.43 (ES: 0.01)); diabetes: attention: F (1,398) = 3.47 (ES:0.01); visual-spatial: F (1,398) = 1.18 (ES: 0.003)). No interaction effects were found. For memory index, no significant differences were found for hypertension and diabetes respectively, though non-diabetic and non-hypertensives had higher mean scores than others. Hypertension: (memory index: F (1,398) = 0.84 (ES: 0.002; Mean: hypertensives = 38.08, non-hypertensives = 40.00) and diabetes: (memory index: F (1,398) = 2.15 (ES: 0.005, diabetics = 36.60, non-diabetics = 40.02). No interaction effects were found. We also showed no significant effects of dementia history and clinical depression on attention and visual spatial index. Dementia history: attention index: F (1,196) = 0.10 (ES:0.001); visual-spatial index: F (1,196) = 0.49 (ES: 0.003)) ; depression: attention index: F (1,196) = 2.69 (ES:0.01); visual-spatial index: F (1,196) = 0.82 (ES: 0.004)). No interaction effects were found. On processing speed and executive function, we found no significant and interaction effects except that of depression on executive function. For dementia history: processing speed: F (1,196) = 0.39 (ES:0.002); executive function: F (1,196) = 0.83 (ES: 0.004)); depression: processing speed: F (1,196) = 2.53 (ES:0.01); executive function: F (1,196) = 4.12 (ES: 0.02, Mean: depressed = 39.34, not depressed = 41.23). No interaction effects were found.

**Figure 3.**
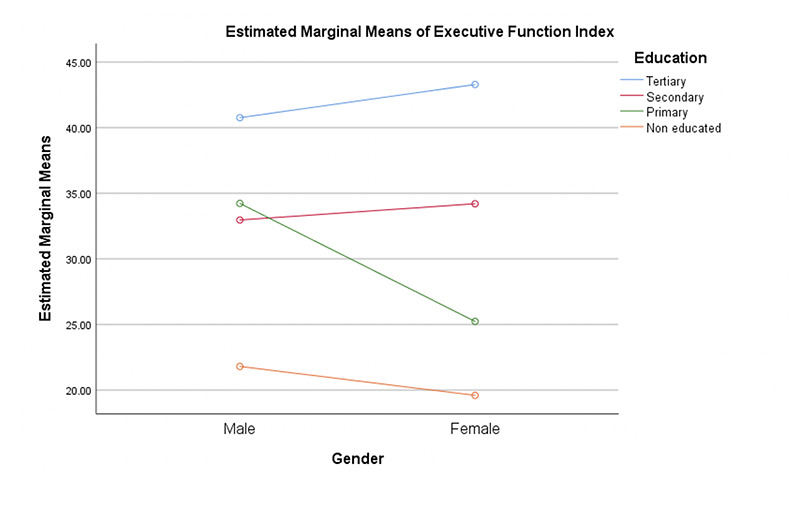
Interaction of education on executive function index

For the memory domain, no significant differences were found for dementia history and depression respectively, though individuals high on depression scale had higher mean scores than others; dementia history: memory index: F (1,196) = 0.07 (ES: 0.001); depression: memory domain: F (1,196) = 0.003 (ES: 0.001; high on depression scale = 39.94, low on depression scale = 40.02). No interaction effects were found. We evaluated the effects of gender and education on global cognition using the MoCA. Our findings showed significant effect of education, F (2,194) = 5.96 (ES: 0.06; Tertiary = 21.47, secondary = 19.18 , Primary = 20.31 ). The pairwise comparison showed significant difference between tertiary education and other two levels of education. No significant gender, F (1,196) = 1.30 (ES: 0.007) and interaction effects, F (2,194) = 1.48 (ES: 0.02) were found. We further showed significant effect of hypertension on MoCA, F (1,196) = 8.52 (ES: 0.04; Mean: hypertensives = 21.34, non-hypertensives = 19.73), but no significant difference was found on diabetes, F (1,196) = 0.05 (ES: 0.001) as well as no interaction of hypertension and diabetes, F (1,196) = 2.64 (ES: 0.01).

## Discussion

We were able to demonstrate prevalence of cognitive impairment in an older adult sample from our community population. To the best of our knowledge, this is the first study in sub-Sahara Africa to investigate the prevalence of cognitive impairment using composite scores derived from multi domain neuropsychological assessment. Previous studies have used single cognitive tools measuring global cognition. Among the tools used were the Mini Mental State Examination and Community Screening Instrument for Dementia (CSID). We calculated the cognitive impairment severity index (CISI) by summing up the performance of participants on the 5 cognitive domains. CISI was classified into normal, borderline, mild and severe cognitive impairments based on performance using 1 and 1.5 SD from the mean respectively. This model represents actuarial neuropsychological criteria as proposed by Jak & Bondi and colleagues (20-21) for the MCI and dementia diagnoses.

Thirty four percent of our sample (representing 150 out of 441 participants) had borderline cognitive impairment, 12.90% (57/441) had mild cognitive impairment while 3.40% (15/441) were severely impaired and 47.90% (219/441) had normal cognition. Taken together, the data indicates that 50.30% of the participants fell into one or more levels of cognitive impairments. This suggests a high prevalence of cognitive impairment and probable dementia in our sample. There were differences between our study and results of other studies reported in Nigeria. For example, while our study shows 3.40% prevalence of severe cognitive impairment, Ocha and Thatcher (11) were reporting 6.40% in northern Nigeria and Baiyewu and colleagues (7) in south-west Nigeria were reporting 6.61% prevalence. Conversely, while we reported 34% borderline cognitive impairment and 12.40% mild cognitive impairment, Baiyewu at.al., (7) were reporting 62% of cognitive impairment without dementia in southwest Nigeria. The difference in outcome between our study and few others (7, 11) was the use of multi domain composite score and stricter cut-off point. Previous studies predominantly used a single measure of global cognition with a mean criteria for determining cut-off levels. Composite scores based on multi domain cognitive assessment, done in this study, reduce the chances of making Type I Error given increased sensitivity and specificity as compared with a single measure of global cognition. Also, the use of 1.5 SD as cut-off point would be more sensitive and specific than the use of mean scores. Use of mean score for clinical evaluation more importantly in neuropsychological assessment would significantly affect test specificity and predictive value. Another plausible explanation for the difference between our finding and that (7) could be nature of assessments used for the two studies. Baiyewu and coworkers (7) included informant and physician interview with low weighted neuropsychological test while our study did not include informant and clinician interview but had high weighted neuropsychological tests.

**Table 4.** Multiple Comparisons of Education on Visual spatial and Attention Domains for Education and Gender

Dependent Variable	(I) Education	(J) Education	Mean Difference (I-J)	Std. Error	Sig.	95% Confidence Interval	
						Lower Bound	Upper Bound
Visual spatial index	Tertiary	Secondary	.5556	.80738	.925	-1.7114	2.8225
		Primary	2.7069*	.73621	.004	.6398	4.7740
		Non educated	5.1556*	.86001	.000	2.7409	7.5702
Attention	Tertiary	Secondary	3.8683*	.82321	.000	1.5569	6.1796
		Primary	5.6327*	.75065	.000	3.5251	7.7403
		Non educated	9.5266*	.87687	.000	7.0646	11.9886
Executive Function Index	Tertiary	Secondary	8.5746*	1.62062	.000	4.2894	12.8598
		Primary	12.7228*	1.46799	.000	8.8412	16.6043
		Non educated	22.2026*	1.69268	.000	17.7269	26.6783
	Secondary	Tertiary	-8.5746*	1.62062	.000	-12.8598	-4.2894
		Primary	4.1481*	1.53526	.042	.0887	8.2076
		Non educated	13.6280*	1.75134	.000	8.9972	18.2588
	Primary	Tertiary	-12.7228*	1.46799	.000	-16.6043	-8.8412
		Secondary	-4.1481*	1.53526	.042	-8.2076	-.0887
		Non educated	9.4798*	1.61114	.000	5.2197	13.7400
Processing Speed Index	Tertiary	Secondary	1.7667	.86402	.225	-.5179	4.0513
		Primary	-1.4519	.78265	.329	-3.5213	.6176
		Non educated	-2.0355	.90244	.139	-4.4217	.3507
	Secondary	Tertiary	-1.7667	.86402	.225	-4.0513	.5179
		Primary	-3.2185*	.81851	.001	-5.3828	-1.0542
		Non educated	-3.8022*	.93371	.000	-6.2711	-1.3333
	Primary	Tertiary	1.4519	.78265	.329	-.6176	3.5213
		Secondary	3.2185*	.81851	.001	1.0542	5.3828
		Non educated	-.5836	.85897	.984	-2.8549	1.6876
	Non educated	Tertiary	2.0355	.90244	.139	-.3507	4.4217
		Secondary	3.8022*	.93371	.000	1.3333	6.2711
		Primary	.5836	.85897	.984	-1.6876	2.8549

We were also able to characterize the performance of our participants on multiple domains of cognition. We presented the percentage of participants that fell below the mean scores by 1 and 1.5 SDs from the mean on 5 cognitive domains respectively. Using the mean score criterion for cut off, processing speed domain had the highest percentage that fell below the mean score followed by executive function, attention/concentration, memory and visual spatial domains. Using a stricter cut-off level (1.5 SD), processing speed had the highest percentage of participants falling below 1.5 SD, followed by visual spatial, memory, attention/concentration and executive function domains. When we translate the mean percentages into actual numbers, it shows that among 441 older adults studied, 217 had problem with attention/concentration, 215 had problem with memory, 183 had problem with visual spatial, 284 had problem with processing speed and 249 older adults had problem with executive function. On 1.5 SD, 23 older adults had problem with attention/concentration, 30 had problem with memory, 57 had problem with visual spatial and 12 had problem with executive function. The above finding provides evidence of the prevalence of cognitive impairments at two levels of cut-offs. The use of mean score cut-off could represent evidence of cognitive impairment at general level of epidemiology while the 1.5 SD represents evidence of cognitive impairment at a clinical level. In neuropsychology clinics, cut-off of 1.5 SD suggests significant impairment that warrants referral to a memory clinic. Using estimation of 1000 participants, our result would show that per 1000 older adults within our community of interest, and on a mean score cut-off, 492 among them would have problem with attention, 487 with memory, 415 with visual spatial, 644 with processing speed and 565 with executive function problem. On 1.5 SD per 1000 older adults, 52 among them would have problem with attention/concentration, 68 with memory, 129 with visual spatial and 27 with executive function problem. This again suggests overwhelming evidence of impairments on domains of cognition in our sample. We found a fair amount of memory impairment, but still less than other studies generally have reported. A point of discussion is on how we classified memory. In this study memory domain was measured using the Craft Story Test (Immediate and delayed). Participants with memory impairment in this population will be higher if we had included visuo-spatial memory as measured by the Benson Design Delayed which could go for visual memory. Given the fact that our participants performed very poorly on Benson Design Delayed, it is a pointer that memory impairment could rank higher if we had classified memory domain using this formular.

The finding is very important towards our understanding of dementia prevalence in Nigeria. Cognitive domain impairments are among hallmarks of AD and dementia in clinical and community settings. They appear to be the last symptom presentation following underlying neurobiological mechanisms in dementia. The presence of cognitive impairments suggests strong neurobiological underpinnings within an individual. To our knowledge this is first study in Nigeria to examine cognitive impairments in older adults using multi domains of cognitive performance while characterizing participants on both mean score and standard deviation from the mean. Majority of studies (7-9) have categorized individual into cognitively impaired and non-cognitively impaired without giving detailed description of the domains of cognitive impairment. In the present study we were able to show how older adults presents on various domains of cognition. It is striking to note the changes in cognitive impairment presentation as we move from mean score cut-off to 1.5 SD cut-off. For example, at mean score level, processing speed and executive function domains had highest percentage while at 1.5 SD representing clinical level assessment, the visuo-spatial domain came up and the executive function was the lowest. This suggests that speed of deteriorations in domains of cognition differ. Our study suggests that older adults present general problem in executive function but would not deteriorate significantly to clinical level. However, they could show less general problem on visuo-spatial domain but could deteriorate faster on this domain. It is interesting to note that visuo-spatial domain was more affected because of participants’ poor performance on the Benson Design Delayed task, signifying underlying visual memory problem. It suggests that visuo-spatial domain presenting greater impairment in our sample at 1.5 SD cut-off is determined by visual memory.

We were able to show significant gender and education differences on visuo-spatial and attention domains in our sample. Our study shows that male older adults performed better than their female counterparts on the two cognitive domains and more educated individuals performed better, all of which might be expected. In addition, there were significant interaction effects of education and gender on visual spatial, attention and executive function domains respectively. From the interaction, less educated females were the most affected group overall. Our study strongly suggests that being female with less education are risk factors for impairment in these three cognitive domains. We found no significant differences in histories of hypertension or diabetes on the 5 cognitive domains. This is contrary to other studies showing differences in cognitive scores in those with hypertension and diabetes. One reason for this result could be the way we categorized hypertension and diabetes. Participants were categorized based on self -report. No objective measures of blood pressure and sugar were taken to confirm their self-report at this time. There is very high possibility that some clinically hypertensive and diabetic participants may have been misclassified. That said, our findings showed differences in memory performance among hypertensives and diabetes with normal participants performing better. Also, we found that participants that reported higher symptoms of depression performed worse on measures of executive function. We also found a significant effect of education on MoCA scores as a measure of global cognition with participants having tertiary education performing better than others. This is in line with previous studies on the effects of education on global cognition (18, 22-23). Equally, hypertensive participants significantly performed worse than non-hypertensives on MoCA, suggesting broader difficulties in this group. Ucheagwu and colleagues (18) also showed systolic blood pressure as predictor of performance on MoCA in middle age adults from Nigeria.

### Limitations of the Study

There are some limitations in the study. First, participants’ diagnoses on hypertension and diabetes were based on self-report. There is likelihood of under diagnoses in the population and that may account for our findings on differences among hypertension and diabetes respectively on cognitive domains. Second, we did not adjust for education on the cognitive severity index. Though there are few educational differences on cognitive domains, adjusting for education level while constructing the cognitive severity index would account for such variations. Our study was an epidemiological survey of community sample, there could be tendency of under reporting of cognitive impairment because we did not use clinical samples. Future, studies are encouraged to compare clinical samples and community population.

## Conclusion

Our study suggests high prevalence of cognitive impairment in older adult population from Nigeria. We were able to show the prevalence of cognitive domains at a general level (mean score cut-off) and at clinical level (1.5 SD) with different cognitive domains assuming dominance at each level. We further showed significant interaction of gender and education on cognition with females being affected the most.
